# Supplementary material for: Optimization of a Protocol for Protein Extraction from Calcified Aortic Valves for Proteomics Applications: Development of a Standard Operating Procedure
Source: Proteomes. 2022 Sep 1;10(3):30. doi: 10.3390/proteomes10030030 (PMC9505568; doi:10.3390/proteomes10030030)
Supplement: Supplementary file 1 [file proteomes-10-00030-s001.zip › Figure S1.pdf]

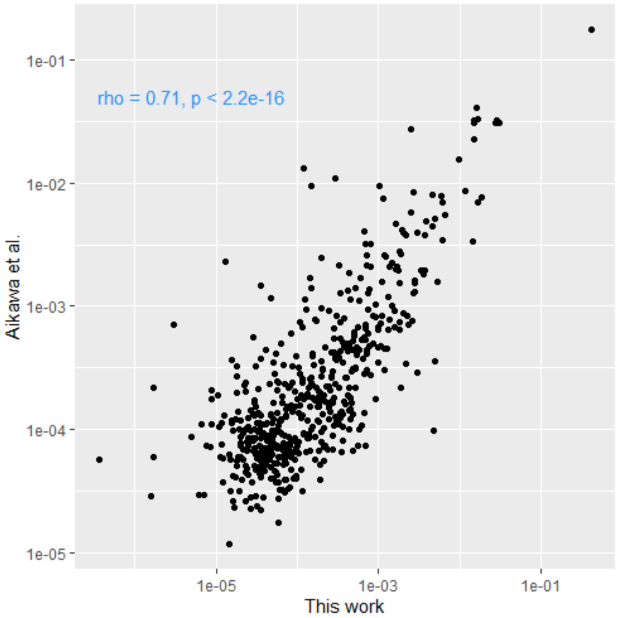

Figure S1. Association between the relative amount (%) of the common proteins between our dataset and Aikawa's *et al*. A Spearman's test was applied, showing a strong and very significant correlation between the two datasets.
