# Supplementary material for: Optimization of a Protocol for Protein Extraction from Calcified Aortic Valves for Proteomics Applications: Development of a Standard Operating Procedure
Source: Proteomes. 2022 Sep 1;10(3):30. doi: 10.3390/proteomes10030030 (PMC9505568; doi:10.3390/proteomes10030030)
Supplement: Supplementary file 1 [file proteomes-10-00030-s001.zip › Figure S2.pdf]

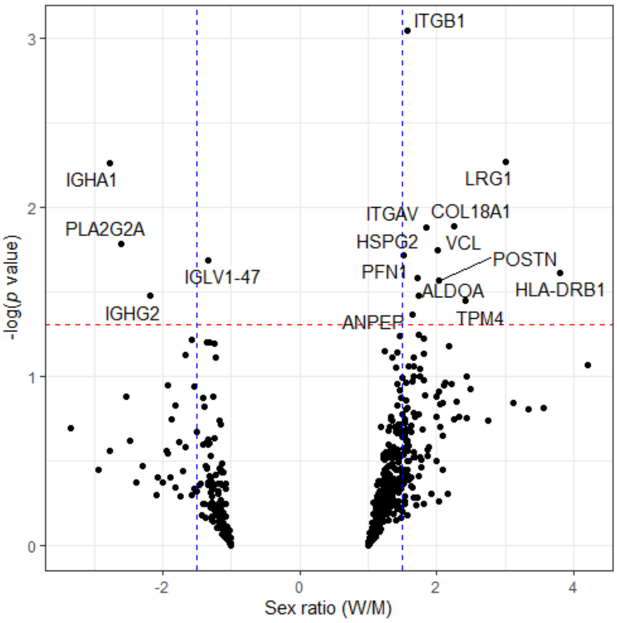

Figure S2. Volcano plot evidencing the proteins differentially expressed in the aortic valves with sex. The red dashed line sets the significance threshold ($p < 0.05$). The blue dashed lines define a fold-change (ratio of percentages) above 1.5 (or below -1.5). All significantly changed proteins are labelled with the respective gene name. All proteins but IGLV1-47 showed a fold-change > 1.5.
