# Supplementary material for: Optimization of a Protocol for Protein Extraction from Calcified Aortic Valves for Proteomics Applications: Development of a Standard Operating Procedure
Source: Proteomes. 2022 Sep 1;10(3):30. doi: 10.3390/proteomes10030030 (PMC9505568; doi:10.3390/proteomes10030030)
Supplement: Supplementary file 1 [file proteomes-10-00030-s001.zip › Supplementary File S1.pdf]

Index

Introduction 2
Aims & scope 2
Abbreviations 2
Consumables 2
Reagents 3
Materials & equipment 3
Protocol 4
Part 1 – Aortic valve processing 4
Part 2 – Aortic valve homogenization and protein extraction 5

Introduction

This protocol is the standard procedure used in UnIC – Cardiovascular Research & Development Centre for the processing, homogenization and protein extraction from aortic valves. A state-of-the-art bead-beating system with zirconium dioxide beads is used to fragment aortic valves, in conjunction with a commercially available RIPA (radio-immunoprecipitation assay) lysis buffer, enriched with phosphatase and protease inhibitors, which promotes cell lysis and protein solubilization. A centrifugation step ensures the purification of a protein-rich supernatant, and the precipitation of cell debris. Protein concentration is estimated using a detergent-compatible protein assay kit.

Aims & scope

The purpose of this SOP is to standardize the procedures for protein extraction from aortic valves aiming at increasing the reproducibility of downstream analysis, particularly, proteomic analysis, with special emphasis on biomarker discovery, and protein analysis by standard immunoassays, such as western blot or similar techniques.

Abbreviations

Acronym/abbreviation	Definition
AV	Aortic valve
DC	Detergent compatible
EDTA	Ethylenediaminetetraacetic acid
LB	Lysis buffer
NP-40	Nonidet P-40
PhIC	Phosphatase inhibitor cocktail
PBS	Phosphate buffer saline
PIC	Protease inhibitor cocktail
RT	Room temperature
SDS	Sodium dodecyl sulphate
UW	Ultrapure water

Consumables

Name	Company	Reference	Storage conditions
60 mL polypropylene sterile flask	Frilabo	25184	RT
Pasteur pipettes	-	-	RT
12 mL screw tap tubes	DeltaLab	401403	RT

2 mL low temperature freezer vials	VWR	479-0821	RT
Precellys ® Zirconium dioxide beads (2.8mm)	Bertin Technologies	P000926-LYSK0-A	RT
Screw-cap O-ring 2 mL microtubes	Sarstedt	72.694.006	RT
Standard 1.5 mL microtubes	-	-	RT
15 mL Falcon tubes	-	-	RT

Reagents

Name	Company	Reference	Storage conditions
Custodiol-CE Bretschneider HTK solution,	Dr. Franz Köhler Chemie Pharmaceuticals GmBH	-	4 ºC
Ice	-	-	-
70% ethanol	-	-	RT
PBS	-	-	4 °C
RNAlater™	Sigma	R0901-500ML	RT
Liquid nitrogen	-	-	Container
Formol	-	-	RT
UW (Simplicity® Water Purification System)	Merck - Millipore	-	RT
RIPA lysis and extraction buffer	Thermo Fisher Scientific	89900 or 89901	4 °C
Halt™ PIC, EDTA-Free 100x	Thermo Fisher Scientific	78439	4 °C
PhosSTOP™ (PhIC)	Roche	4906837001	4 °C
EDTA disodium salt dihydrate (≥99%)	Merck - Millipore	1084540100	RT
DC Protein Assay kit	Bio-Rad	5000116 or 5000120	RT

Materials & equipment

Name	Notes
Petri dish	
Precision scale	Maximal mass difference ± 0.01 g
Analytical scale	Maximal mass difference ± 0.1 mg
Dissection set (scissors and tweezers)	To cut the valve leaflets

Rack	To hold the tubes
Liquid nitrogen container	To flash-freeze the samples
Vortex	
Mortar and pestle	~12cm diameter mortar
Spatulas and tweezers	
Bead-beating system, Bertin Instruments	The protocol has been optimized for Minilys (P000673-MLYS0-A), but it is scalable to the Precellys products
Chronometer	
Benchtop centrifuge	Refrigerable, compatible with 2 mL and 1.5mL tubes, and reaching 12,000 rpm
Minicentrifuge	To spin down lysates

Protocol

Part 1 – Aortic valve processing

1. Collect the resected aortic valve to a pre-chilled (4 ºC) 60-mL flask half-full with sterile Custodiol.
2. Transport the samples on ice to the laboratory.

   The samples should be kept on ice until all material is at hand.
3. Turn the sterile hood on.
4. Disinfect the bench with 70% ethanol and get the materials ready to use (vials, rack, Petri dish, dissection tools, mortar, and pestle).
5. Spray and disinfect the flask with 70% ethanol and transfer it to the hood.
6. Place a styrofoam box with ice at hand.
7. Label some 2 mL low-temperature freezer vials with the respective sample code. Indicate the sample type (aortic valve), the respective code, and whether the vial contains RNA-later.
8. Disinfect a Petri dish and transfer it to the sterile hood.
9. Place the valve in the dish and wash thrice with PBS, using the disposable Pasteur pipettes.

   IMPORTANT: If the sample is to be used for cell isolation and culture protocols, use filtered PBS and work under aseptic conditions.
10. [**OPTIONAL**] – Cut a representative (cross-sectional) fraction of the aortic valve from the annulus to the center (anatomical orifice) for cell culture. Transfer such a fraction to a new flask half-full with sterile Custodiol and keep it on ice until further processing.
11. [**OPTIONAL**] - Cut a representative (cross-sectional) fraction of the aortic valve from the annulus to the center (anatomical orifice) for histology. Transfer such a fraction to a pre-labeled 12-mL flask containing formol.

The sample should be in at least 8 volumes of formol.

12. [**OPTIONAL**] – Cut a non-thick fragment (maximum of 0.5 cm in any – not all - of the dimensions) and transfer it to a pre-labeled RNA later-containing vial. Keep the vial on ice.

    The sample should be in at least 5-10 volumes of RNA later.

13. Cut the remaining sample into fragments small enough to fit the vials. Tightly close the lids and immediately flash freeze the samples in the liquid nitrogen canister.

    These samples will be used for Part 2 of the protocol.

14. [**OPTIONAL**] – Maintain samples in RNA later at 4 ºC, overnight, to allow a full permeation of the sample. After one to three days (maximum), transfer the tubes to the -80 ºC freezer.
15. Discard the sample containers and the disposable pipettes to waste.
16. Tie the waste bag and discard it in the waste bin.
17. Disinfect the waster container, the rack used to hold the vials, and the bench by spraying with 70% ethanol.
18. Wash the Petri dish and disinfect with 70% ethanol.
19. Run a 30-min UV disinfection cycle on the hood.
20. Save the samples for proteomics (step 13) in the -80 ºC freezer, until further processing.
21. Turn the hood off.

Part 2 – Aortic valve homogenization and protein extraction

22. Label one O-ring 2 mL tube, and at least three 1.5 mL tubes (one for centrifugation, one for quantification and another for storage) for each sample.

    Should you wish to aliquot the sample, label more tubes.

23. In another set of 2 mL tubes weigh the zirconium dioxide beads. The amount should be 40x the amount of tissue to homogenize. Fill as many tubes as the number of samples to process. Fill an extra tube with beads, just in case some sample exceeds greatly the initially planned amount.

    This protocol has been optimized for 30 mg of starting material, requiring 1.2g of beads.

24. Fill a styrofoam box with ice. Keep the RIPA buffer, PIC and PhIC on ice.
25. Prepare the lysis buffer (LB): 25 mM Tris-HCl pH 7.6, 150 mM NaCl, 1% NP-40, 1% sodium deoxycholate, 0.1% SDS, 1 mM EDTA, enriched with protease and phosphatase inhibitors:

*The following sub-steps are designed for a total of **10 mL lysis buffer**, which is enough to process ~30 AV samples weighing around ~30 mg, or ~ 20 samples weighing around ~ 50 mg.*

25.1. In an empty 15mL Falcon tube, weigh 3,7 mg of EDTA.

25.2. Add a PhIC tablet.

Each tablet should be dissolved in 10 mL of solution. It is recommended to prepare the lysis buffer in multiples of 10 mL.

25.3. Add 9.9 mL of cold RIPA to the tube.

25.4. Vortex until all reagents are solubilized.

For 10 mL, this step takes less than one minute.

25.5. Add 100 µL of cold PIC.

25.6. Close the lid, and homogenize by gently inverting ten times.

25.7. Keep the LB on ice.

26. Refrigerate the centrifuge to 4 °C.
27. Fill a container with liquid nitrogen.
28. Disinfect the lab bench around the scale with ethanol 70%.
29. Disinfect the mortar, pestle, and tweezers with ethanol 70%.
30. Take one sample vial, at a time, from the freezer and place it immediately on ice.
31. Tare the respective O-ring tube, lid off, in the analytical scale.
32. Add some nitrogen to the mortar and cool the pestle.
33. Quickly transfer the sample to the mortar and break it down.
34. After obtaining a piece of ~30mg, transfer the remaining sample back into the cryotube, and place it again on ice.

    30 mg of tissue corresponds roughly to 8-9mm x 8-9mm pieces.
35. Add more nitrogen to break into smaller pieces of 2-3 mm and transfer them to the O-ring tube. Register the weight.

    Do not over-powder the sample, as this leads to major sample loss and to quick defrosting.
36. Add the LB and using the pipette tip immerse any dry tissue fragments attached to the tube wall.

    The LB is easily calculated as 10 to 20 times the amount of tissue, e.g., for 30 mg, 300 to 600 µL of the buffer is required. The LB/tissue mass ratio will depend on the downstream application, i.e., whether samples should be preferably more concentrated (e.g., for gel-based approaches). For standard proteomics (shotgun), 10 µL/mg is enough. When the amount of protein is more important, for instance, when some enrichment step is to be performed (e.g., phosphopeptide enrichment, co-immunoprecipitations, etc…) 20 µL/mg is preferable.
37. Add the corresponding beads. For every 2 mg of additional tissue, add one extra bead (~ 0.07g). Close the lid and quickly place the tube on ice.
38. Tare the next O-ring tube.

39. Store the remaining unprocessed sample back in the freezer. Take the next sample and keep it on ice.
40. Repeat steps 32-38 and proceed until all samples have been homogenized.

    Since some AVs are harder than others to break down on nitrogen due to the varying content in calcium, some samples take longer to process. For that reason, it is preferable to keep them in the freezer and take one at a time to prevent unwanted defrosting.
41. Homogenize the samples in the Minilys instrument, at maximum speed (5,000 rpm), for 30 seconds.
42. Cool the samples down on ice for 5 minutes.
43. Repeat step 41.
44. Spin down the samples for one minute using the minicentrifuge.

    If there is still too much foam in some samples, repeat this step in 30 seconds increments.
45. Transfer the supernatant to an empty 1.5mL tube.
46. Centrifuge the sample at 12,000 rpm (13,680 × *g*) for 15 minutes at 4 °C.
47. Transfer the supernatant to an empty 1.5mL tube. Discard the pellet.
48. Save 10-25 µL for quantification.
49. Aliquot the sample in 100 µL fractions, and store them at -80 ºC.
50. Save the remaining LB at 4 °C for the preparation of the protein standards, and sample dilutions for quantification on the same or the next day.
51. Estimate the protein concentration using the DC assay, following the manufacturer's instructions.

    The expected protein concentrations range between 1.5-3 µg/µL.

    NOTE: The DC assay is not compatible with urea-rich buffers. Thus, when protein extraction is performed with a urea buffer, the sample aliquots for quantification should be precipitated with acetone and resolubilized in 1% SDS. To do that, simply add 9 volumes of ice-cold acetone to each sample and let the protein precipitate overnight at -20 ºC. In the next day, centrifuge the samples for 30' at 12,000 × g at 4 ºC, discard the supernatant and resuspend in 1% SDS before proceeding with the manufacturer's protocol.
